# Absence of Left Circumflex Artery: A Rare Congenital Disorder of Coronary Arteries

**DOI:** 10.1155/2017/8710135

**Published:** 2017-04-16

**Authors:** Saad Ullah, Muzammil Khan, Noman Ahmed Jang Khan, Hassan Zeb, Roshan Patel

**Affiliations:** Department of Internal Medicine, Temple University/Conemaugh Memorial Hospital, 1086 Franklin Street, Johnstown, PA 15905, USA

## Abstract

Congenital absence of left circumflex artery is a rare occurrence and very few cases have been reported in literature. It is a benign incidental finding; however some patients present with sudden onset chest pain mimicking acute coronary syndrome often resulting in detection of this rare anatomy on coronary angiography. Coronary computed tomography angiography is a relatively new noninvasive imaging modality which can be used to confirm this suspicion and diagnose this unique morphology reliably.

## 1. Introduction

Coronary artery anomalies are the second most common cause of sudden cardiac deaths among young athletes, with an overall prevalence of about 0.3% to 5.6% among the general population [1]. Congenital absence of left circumflex artery (LCX) is a rare anatomical defect which is invariably associated with right dominant circulation. Patients with congenital absence of LCX can present with variable symptoms ranging from dyspnea on exertion to acute onset myocardial infarction. In this article, we describe a case of a 58-year-old female presenting with exertional dyspnea who underwent stress test which was positive for ischemia in left anterior descending (LAD) territory. Coronary angiography was suspicious for absent LCX which was later confirmed with the gold standard, coronary computed tomography angiography (CCTA).

## 2. Case Presentation

**Figure 1:**
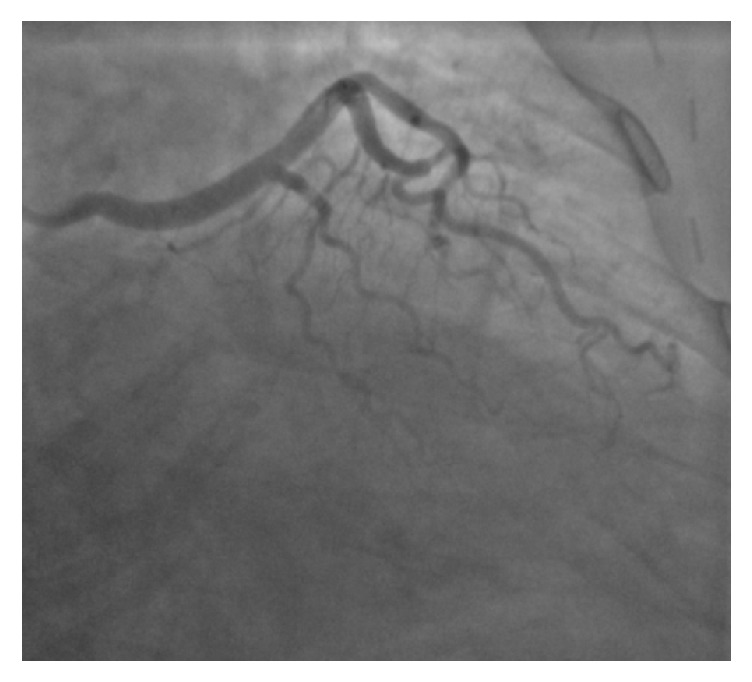
RAO caudal view showing absent left circumflex (LCX) and long left main coronary artery.

**Figure 2:**
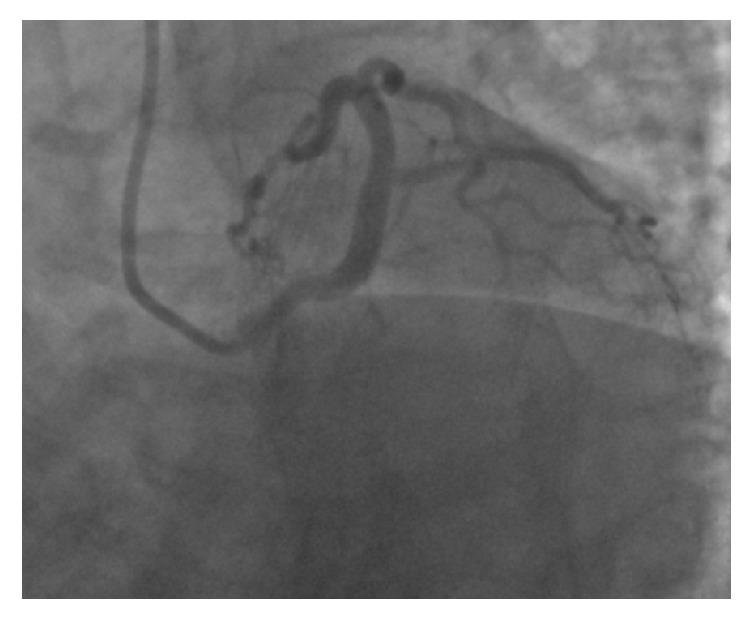
LAO caudal view showing absent left circumflex (LCX).

**Figure 3:**
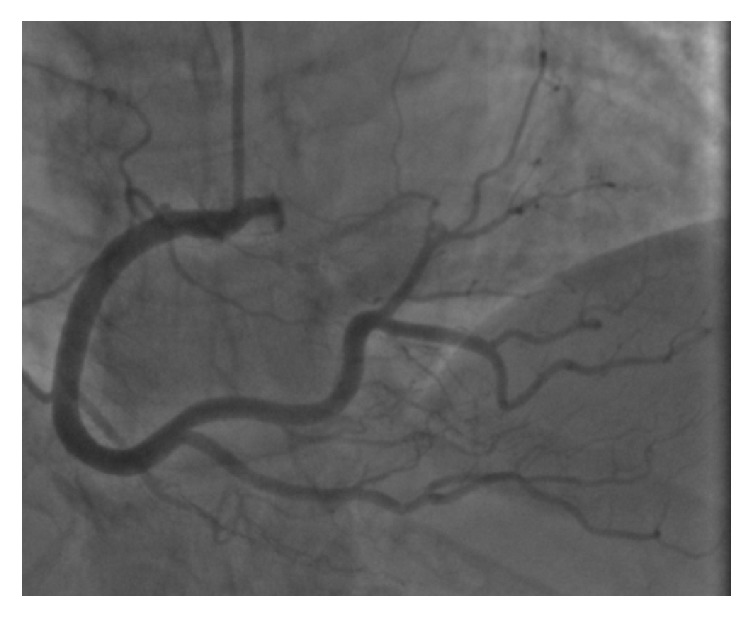
AP cranial view showing dominant RCA supplying LCX territory.

**Figure 4:**
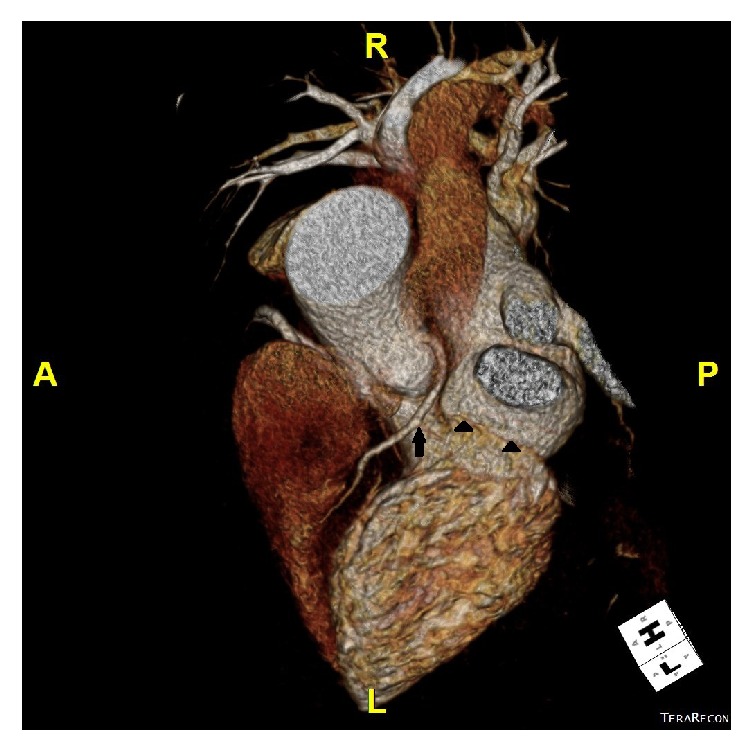
Top view three-dimensional computed tomography scan showing single left anterior descending artery (arrow) and absence of LCX in the atrioventricular groove (arrow heads).

**Figure 5:**
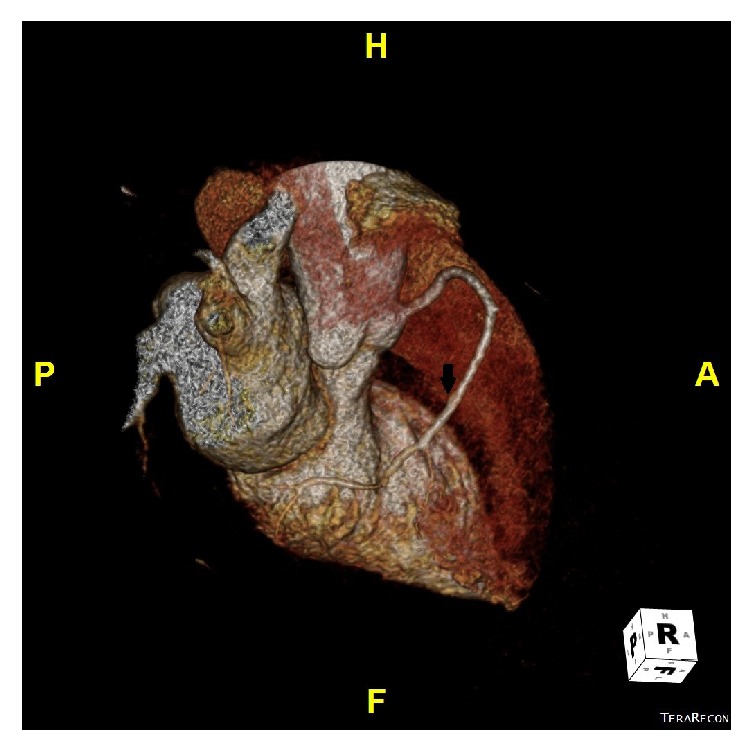
Inferior view three-dimensional computed tomography scan showing large dominant right coronary artery (arrow).

A 58-year-old Caucasian female presented to the emergency department from her primary care physicians office for the evaluation of exertional dyspnea and chest tightness for the last six months. She had remote history of hypertension, gastroesophageal reflux disease, and hypothyroidism. Physical examination revealed a well-nourished obese female with a pulse of 56 beats per minute, blood pressure of 118/72 mm Hg, respiratory rate of 18 breaths per minute, and peripheral arterial oxygen saturation of 96%. Cardiovascular examination revealed regular S1 and S2 heart sounds with no additional heart sounds or murmurs. Initial 12-lead electrocardiogram revealed sinus bradycardia, normal axis with no ischemic changes. Routine blood work including cardiac enzymes and chest radiography was also within normal limits. Transthoracic echocardiogram revealed normal left ventricular systolic function with ejection fraction of 65% with mild mitral and tricuspid regurgitation. Pharmacologic stress test with nuclear imaging revealed medium size area of reversible moderate intensity ischemia in the base to the distal anterior wall suggestive of significant stenosis of LAD territory. Coronary angiography revealed a long left main (Figure 1), normal LAD and absence of LCX with super-dominant RCA (Figures 2 and 3 and video 1; see Supplementary Material available online at https://doi.org/10.1155/2017/8710135) and no obstructive lesion of the coronaries. Left ventriculography was normal with an ejection fraction of 55%. Aortography was also performed which did not reveal any anomalous origin of LCX. CCTA was ordered to confirm the suspicion which revealed absence of left circumflex artery (Figure 4) and a right dominant circulation (Figure 5). CT pulmonary angiogram was also performed which failed to reveal anomalous origin of LCX from pulmonary arteries.

## 3. Discussion

Congenital absence of left circumflex coronary artery is an extremely rare anomaly with a reported incidence between 0.003% and 0.067% [1]. More commonly encountered anomalies involving this artery include LCX originating from proximal right sinus of valsalva, often sharing a common ostium with RCA, or as a proximal branch of right coronary artery [1]. Complete absence results from agenesis of LCX in the left atrioventricular groove. In this condition, lateral wall of the left ventricle is supplied by a super-dominant right coronary artery or occasionally by a multiple diagonal branches of LAD [1, 2]. It is an incidental benign finding on coronary angiography. However, it can present with significant clinical symptoms in up to 20% of the cases. Most of the patients present with exertional chest pain. One hypothesis that can explain exertional symptoms is steal phenomenon. This phenomenon results from increased metabolic demands in the LCX territory resulting in ischemic changes in LAD or RCA territories mimicking an acute coronary event [1, 2]. Varela et al. [3] reported a case of a 52-year-old male with congenital absence of LCX who presented with 90% stenosis of super-dominant RCA resulting in inferolateral and posterior wall myocardial infarction. Although anomalous origin of LCX has been associated with accelerated atherosclerosis because of abnormal wall stress, mechanical trauma, and abnormal flow strain, the absence of LCX has not been associated with major cardiac events [3].

The currently available diagnostic modalities for this rare entity include transthoracic echocardiogram (TTE), transesophageal echocardiogram (TEE), cardiac magnetic resonance imaging, coronary computed tomography angiography (CCTA), and invasive angiography. The role of stress test as a screening tool for exertional chest pain is limited in case of coronary artery anomalies [4]. TTE has been compared to invasive angiography in detection of congenital coronary artery anomalies (CCAA). The incidence of CCAA on TTE was reported to be 0.17% as compared to 1.07% with invasive angiography which makes TTE a less favorable screening tool for detection of CCAA and warrants further testing necessary. Based on this incidence of CCAA on invasive angiography, the importance of coronary angiography cannot be undermined, although invasive angiography provides only a two-dimensional image when compared to three-dimensional CCTA [1]. Although cardiac MRI is also a noninvasive imaging modality which does not require use of contrast agent or radiation, its inability to visualize smaller coronary arteries limits its use for CCAA evaluation [1].

CCAA are commonly detected incidentally on invasive coronary angiography or CCTA when the patient is being evaluated for suspected coronary artery disease (CAD). In appropriate clinical settings (symptomatic patients with low to intermediate risk for CAD who cannot exercise or cannot have pharmacologic nuclear stress test or have equivocal stress testing) because of noninvasive nature, safety, rapid acquisition of results, lower radiation exposure, better sensitivity, and more widespread availability CCTA is emerging as a reasonable first-line tool for evaluation of coronary anatomy to rule out CCAA [2, 5, 6]. Recently, American College of Cardiology also included CCTA as a first-line tool for known or suspected anomalies, provided appropriate criteria are used [5, 6]. Though, invasive angiography remains gold standard in patients with high pretest probability of CAD [6]. Ghadri et al. compared CCTA and invasive angiography for prevalence of coronary anomalies. The reported prevalence of coronary anomalies on noninvasive CCTA was 7.85% compared to 2.02% on invasive angiography (*p* < 0.01) which highlights CCTA as an important tool for assessment of suspected coronary anomalies [5]. In a small series of 16 patients, CCTA detected 100% of coronary anomalies as compared to 53% of anomalies on invasive angiography further supporting its use as a better tool for coronary anomalies [5]. In addition, CCTA also offers highly accurate description of coronary anomalies because it can present a three-dimensional image and reliably delineate the origin, course, and termination of coronary arteries and their relationship to cardiac and noncardiac structures [2, 5].

Because CCAA obscures the normal coronary anatomy and increases the risk for accidental damage to the anomalous vessels during bypass surgery and cardiac catheterization, it is imperative to identify CCAA before opting these cardiac procedures [3]. CCAA has been associated with sudden cardiac deaths in young population particularly athletes which highlights the importance of early diagnosis [5].

There is no specific treatment for absent LCX but it is decisive to differentiate 100% occluded LCX from absent LCX to avoid accidental damage to the LCX and choose appropriate revascularization approach if ischemia is detected [3].

## 4. Conclusion

Congenital absence of LCX is an uncommon coronary vasculature pattern often detected incidentally during various cardiac imaging studies, usually on cardiac catheterization or coronary computed tomography angiography. Physicians should be aware and be able to identify this rare entity particularly in patients undergoing bypass surgery to ensure adequate reperfusion of myocardium and to avoid any iatrogenic injuries to these anomalous vessels during these procedures.

## Supplementary Material

Cardiac catheterization showing absence of left circumflex artery.
